# Metabolites-Based Network Pharmacology to Preliminarily Verify In Vitro Anti-Inflammatory Effect of Ardisiacrispin B

**DOI:** 10.3390/ijms242317059

**Published:** 2023-12-02

**Authors:** Wen Zhou, Guixiang Yang, Yushuang Wen, Qian Xiao, Le Sun, Yongjun Li, Zipeng Gong, Yonglin Wang

**Affiliations:** 1 School of Basic Medicine, Guizhou Medical University, Guiyang 550004, China; 2019010010076@stu.gmc.edu.cn; 2 School of Pharmaceutical Sciences, Guizhou Medical University, Guiyang 550004, China; 2021110030958@stu.gmc.edu.cn (G.Y.); 2021110030951@stu.gmc.edu.cn (Y.W.); 3322020833@stu.cpu.edu.cn (Q.X.); 2021110030950@stu.gmc.edu.cn (L.S.); 3 State Key Laboratory of Functions and Applications of Medicinal Plants, Guizhou Provincial Key Laboratory of Pharmaceutics, Guizhou Medical University, Guiyang 550004, China; 4 State Key Laboratory of Functions and Applications of Medicinal Plants, Engineering Research Center for the Development and Application of Ethnic Medicine and TCM (Ministry of Education), Guizhou Medical University, Guiyang 550004, China; liyongjun026@gmc.edu.cn

**Keywords:** anti-inflammatory, ardisiacrispin B, metabolites, network pharmacology, PI3K-AKT

## Abstract

*Ardisiae Crenatae Radix* is an ethnic medicinal herb with good anti-inflammatory activity. Ardisiacrispin B is one of the main components in *Ardisiae Crenatae Radix* extract, with a content of up to 16.27%, and it may be one of the pharmacological components through which *Ardisiae Crenatae Radix* exerts anti-inflammatory activity. At present, reports on ardisiacrispin B mainly focus on anti-tumor effects, and there have been no reports on anti-inflammatory activities. As a triterpenoid saponin, due to its large molecular weight and complex structure, the composition of substances that function in the body may include other forms after metabolism, in addition to compounds with original structures. Exploring the anti-inflammatory effects on the prototypes and metabolites of the compound may provide a more comprehensive response to the characteristics of ardisiacrispin B’s anti-inflammatory action. In this study, ardisiacrispin B was analyzed for metabolites to explore its metabolic processes in vivo. Subsequently, the anti-inflammatory effects of the prototypes and metabolites were further analyzed through network pharmacology, with the expectation of discovering the signaling metabolic pathways through which they may act. Finally, the anti-inflammatory effects of ardisiacrispin B in vitro and the effects on key signaling pathways at the protein level were explored. The results of this study showed that the isolated compounds were confirmed to be ardisiacrispin B. After the metabolite analysis, a total of 26 metabolites were analyzed, and the metabolism process in rats mainly involves oxidation, dehydration, glucuronide conjugation, and others. Speculation as to the anti-inflammatory molecular mechanisms of the prototypes and metabolites of ardisiacrispin B revealed that it may exert its anti-inflammatory effects mainly by affecting the PI3K-AKT pathway. Further anti-inflammatory mechanisms demonstrated that ardisiacrispin B had a good anti-inflammatory effect on LPS-induced RAW264.7 cells and a strong inhibitory effect on NO, TNF-α, and IL-1β release in cells. Furthermore, it had significant inhibitory effects on the expression of PI3K, P-PI3K, AKT, and P-AKT. This study supplements the gaps in the knowledge on the in vivo metabolic process of ardisiacrispin B and explores its anti-inflammatory mechanism, providing an experimental basis for the development and utilization of pentacyclic triterpenoids.

## 1. Introduction

Inflammation, an immune-defense response to harmful stimuli, is a pathological process that reflects the states of many acute and chronic diseases. This response is mainly caused by stimulating macrophages to release inflammatory media such as cytokines, chemokines, growth factors, and others, which have the characteristics of multiple pathways and mechanisms [1,2]. The short-term inflammatory response is a key step in the body’s self-repair and involves multiple responses in different cells and organs. However, prolonged and uncontrolled inflammation can lead to a variety of diseases, such as cardiovascular dysfunction, metabolic disorders, autoimmune diseases, rheumatoid arthritis, heart attacks, cancer, and others. Inflammation can be caused by a variety of factors, such as biological factors, physical and chemical factors, abnormal immune responses, foreign bodies, and tissue necrosis.

The research shows that most of the active ingredients of natural herbaceous plants have anti-inflammatory effects, and this has become one of the important research directions for new anti-inflammatory drugs with strong activities, novel structures, and few toxic side effects. In particular, terpenoids have drawn significant attention due to their good pharmacological actions, which include anti-inflammatory [3], anti-tumor [4], anti-bacterial [5], anti-human-immunodeficiency-virus [6], blood-sugar-lowering [7], liver-preservation [8], and other properties [9,10,11].

*Ardisiae Crenatae Radix* (*Ardisia crenata* Sims plant, *Ardisia* genus, *Primulaceae* family) [12], is a commonly used herbal medicine for ethnic minorities in southwestern China. with good anti-inflammatory activity. Its marketed preparations, Kaihoujian Spray and Qingsangyanhou Buccal Tablets, are mainly used to treat acute and chronic pharyngitis, tonsillitis, stomatitis, and others [13]. Preliminary investigation results showed that both the crude (80% ethanol extract) and refined products (n-butanol—70% ethanol refining) of *Ardisiae Crenatae Radix* have good anti-inflammatory activity, which can inhibit the secretion of NO, TNF-α, and IL-β in lipopolysaccharide (LPS)-induced RAW264.7 cells [14]. In the refined product, bergenin, 11-O-galloylbergenin, and ardisicrispin B account for over 80% of the total solids [15], indicating that these three components may be the main pharmacological substances responsible for the anti-inflammatory effect of the refined product of *Ardisiae Crenatae Radix*. The anti-inflammatory effects of bergenin [16] and 11-O-galloylbergenin [17] have been reported in the literature, but the reports on ardisicrispin B mainly focus on the anti-tumor effect [18], and there are no reports on the anti-inflammatory effect. Therefore, based on the above background, the current paper investigates the anti-inflammatory activity of ardisicrispin B in vitro.

Ardisiacrispin B, a natural saponin compound, belongs to the pentacyclic triterpenoid saponins, which are mostly found in the roots and rhizomes of *Ardisia*-family plants [19,20,21]. It is reportedly a cytotoxic molecule, sensitive to a variety of cancer cells, and cytotoxic to multi-drug-resistant cancer cells [18]. However, its involvement in the inflammatory response has not been reported. As a pentacyclic triterpenoid saponin compound, it is characterized by its large molecular weight and complex structure. However, little research has been reported on its absorption and metabolism, its pharmacological activity, its anti-inflammatory effects, and their mechanisms in vivo. Due to their large molecular weights and complex structures, the compositions of the substances that function in the body may include other forms after metabolism, in addition to compounds with their original structures.

Therefore, the analysis of metabolites in rats was identified in this study at the same time. The prototype and metabolites were used as two parts for network-pharmacology studies of anti-inflammatory effects, and the two parts of the compounds were enriched to obtain a common signaling pathway. Further Western blot tests were carried out to explore the effect of ardisicrispin B on key proteins in the common signaling pathway. The predicted common signaling pathway can not only reflect the anti-inflammatory pathway of ardisicrispin B itself but also take into account the potential anti-inflammatory characteristics of its metabolites. It may be able to more comprehensively map the role of ardisicrispin B.

The process of macromolecular-compound disposal in the body is complex, and most of these compounds are degraded through in vivo metabolism so as to exert pharmacological effects, so it is important to explore their metabolites in vivo. Therefore, in the experiment in this study, we utilized the mechanism of molecular-chain breakage during oral metabolism to derive the structural formulae of the compounds obtained during in vivo metabolism by means of UHPLC/Q Exactive Plus MS and applied these structural formulae to speculate as to the mechanism of ardisiacrispin B in anti-inflammatory processes, by means of a network-pharmacology approach. The lipopolysaccharide (LPS)-stimulated RAW264.7 cell-inflammation model was then used to validate the model and provide a reference point for future research on macromolecular compounds.

## 2. Results

### 2.1. Analysis of Metabolites in Plasma after Administration of Ardisiacrispin B

As shown in Figure S1, the total-ion-current diagram of the mixed plasma samples after the intragastric administration of ardisiacrispin B was obtained and analyzed by Xcalibur 4.1 and Compound Discoverer 3.2. The metabolites of ardisiacrispin B were predicted by using the fragmentation pattern of the prototype components, high-resolution mass-spectrometry data, error values, and related research. The prototype form of ardisiacrispin B was detected in the rat plasma, and 26 metabolites were speculated.

**Figure 1:**
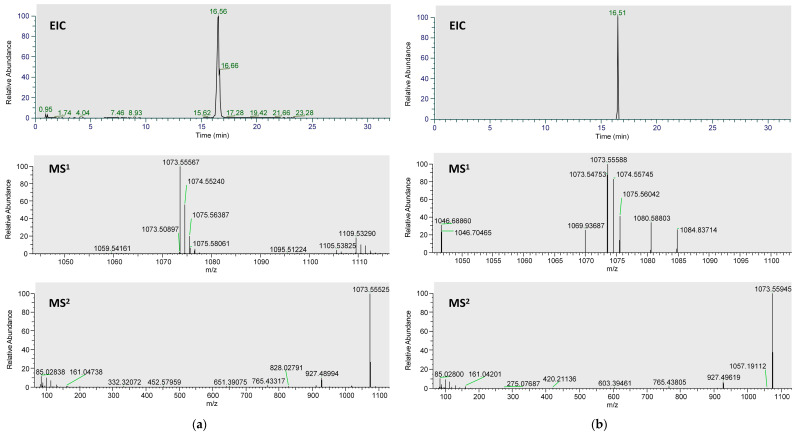
The extracted-ion chromatograms (EICs): primary and secondary mass spectra of ardisiacrispin B in (**a**) reference substances and (**b**) plasma samples.

**Figure 2:**
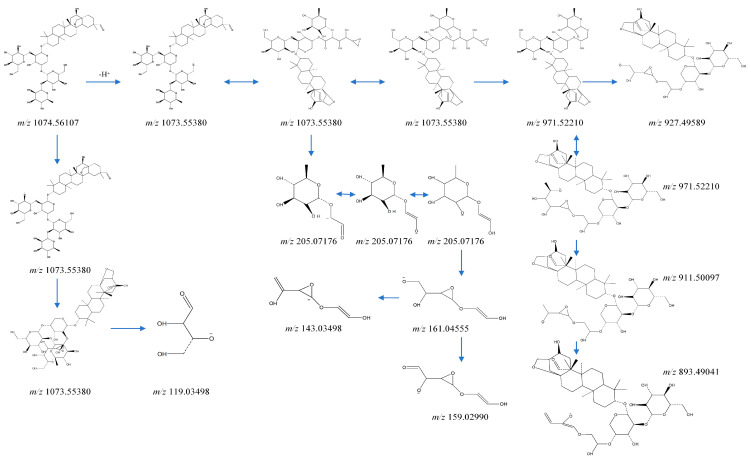
Possible mass-spectrometry cleavage pattern of ardisiacrispin B.

**Figure 3:**
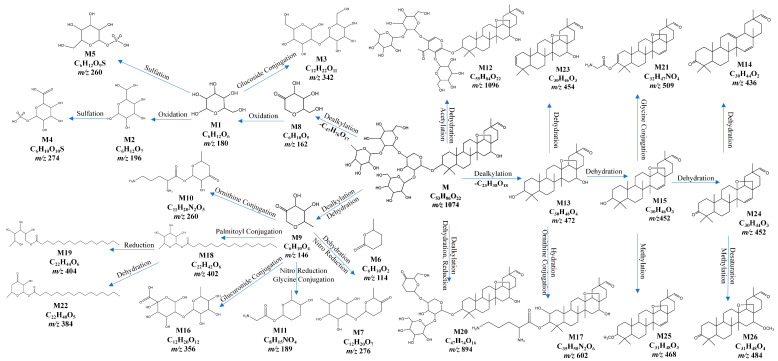
Analysis of metabolites in rat plasma after intragastric administration of ardisiacrispin B (M). The mass-spectra information corresponding to the chemical formulas of the “M” or “M+ numbers” in the figure can be seen in Table 1.

The extracted-ion chromatograms (EICs, Figure 1), primary and secondary mass spectra of ardisiacrispin B in the plasma samples, and reference substances are shown in Figure 1a,b. The spectra of the two are essentially the same, indicating that the ardisiacrispin B prototyped into the blood. The fragmentation-pattern analysis of its mass spectrometry was completed by using Mass Frontiers 8.0, and the results are as follows (Figure 2). Under voltage, ardisiacrispin B undergoes de-H^+^ at the hydroxyl or hydroxymethyl group on the substituted glucose group to form *m*/*z*1073[C_53_H_85_O_22_]^−^. The hydroxymethyl group is charged with O and undergoes charge transfer, ring-opening degradation of the glucose group and the E ring, conversion through a variety of intermediates, the loss of −C_4_H_6_O_3_ and formation of *m*/*z*971[C_49_H_79_O_19_]^−^. The *m*/*z*971 loses -C_2_H_4_O to produce *m*/*z*927[C_47_H_75_O_17_]^−^, which then loses -O to *m*/*z*911[C_47_H_75_O_16_]^−^. Furthermore, *m*/*z*911 undergoes electron transfer, enol interconversion, the ring opening of the cyclic ether, and the loss of one molecule of -H_2_O to produce *m*/*z*893[C_47_H_75_O_16_]^−^. Alternatively, the hydroxymethyl group of glucose is charged to open the ring, electrons are transferred, and m/z205[C8H13O6]- undergoes a dehydration reaction *m*/*z*205[C_8_H_13_O_6_]^−^. Subsequently, it undergoes an enolitic intercalation, in which the sugar group opens the ring and loses the -C_2_H_4_O, becoming *m*/*z*161[C_6_H_9_O_5_]^−^. The *m*/*z*161 may lose a molecule of H_2_O to *m*/*z*143[C_6_H_7_O_4_]^−^ or undergo degradation, losing two molecules of -H to *m*/*z*159[C_6_H_7_O_5_]^−^. The hydroxyl group is charged and undergoes charge transfer; the glucose opens the ring, and -OH is cleaved and transfers electrons to =O to produce *m*/*z*119[C_4_H_7_O_4_]^−^. A total of 26 metabolites were detected, mainly involving metabolic pathways including oxidation, dehydration, reduction, conjugation, and others. It involves one-phase and two-phase metabolism. The specific metabolic process of ardisiacrispin B is shown in Figure 3, and the MS information for specific metabolites is shown in Table 1.

### 2.2. Molecular-Mechanism Analysis of the Anti-Inflammatory Effect of Ardisiacrispin B and Its Metabolites

**Figure 4:**
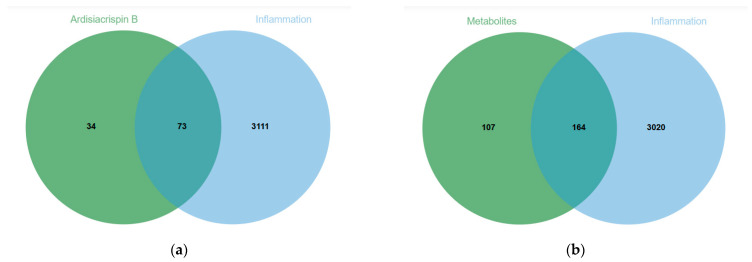

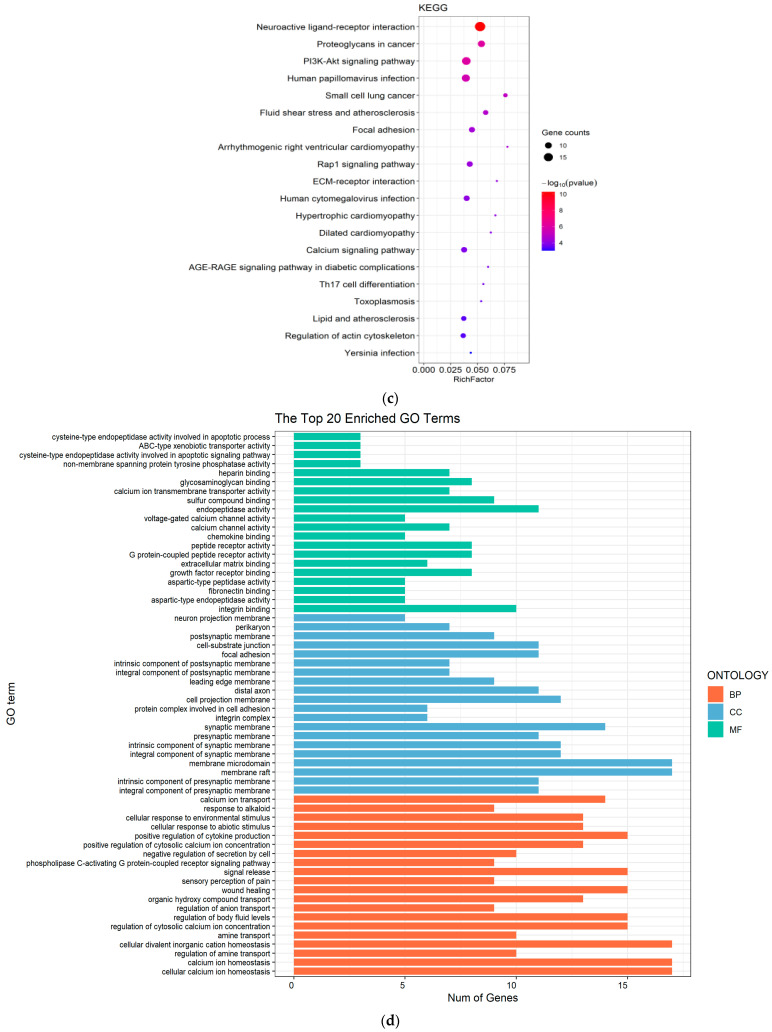

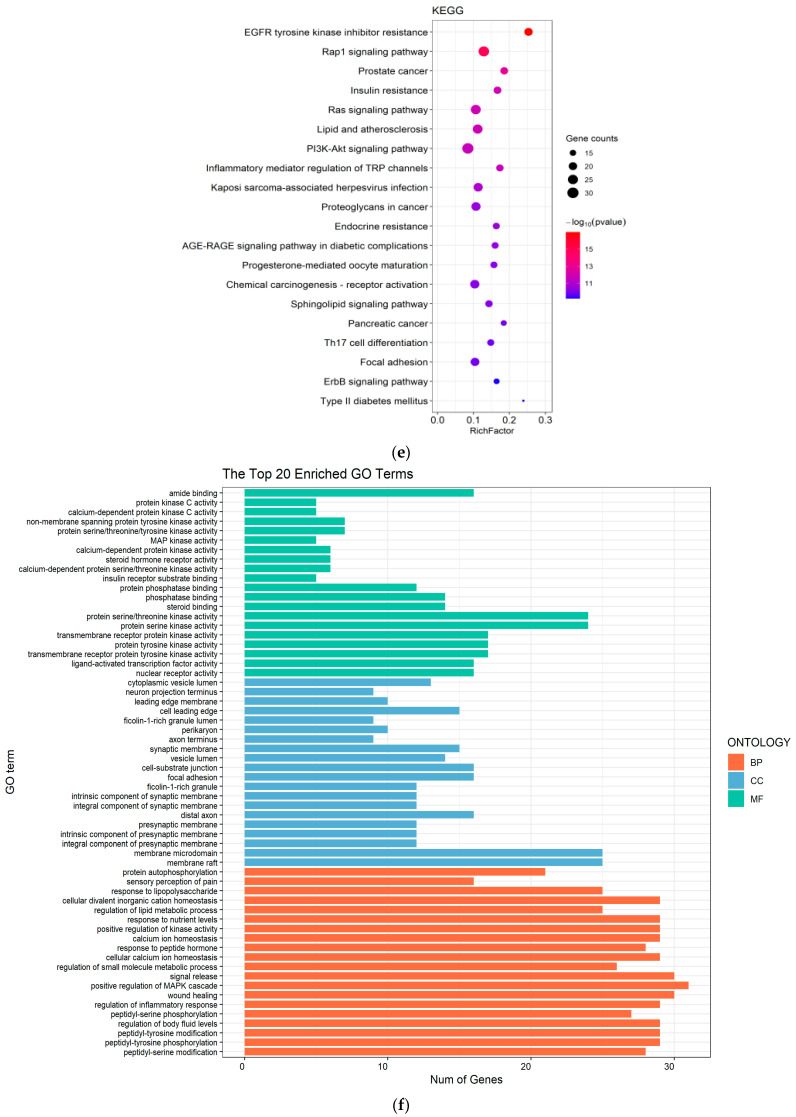
Prediction of molecular mechanism of anti-inflammatory effect of ardisiacrispin B and its metabolites based on network pharmacology: Venn diagram of (**a**) ardisiacrispin B and its (**b**) metabolites; (**c**) KEGG and (**d**) GO analyses of ardisiacrispin B; (**e**) KEGG and (**f**) GO analyses of metabolites.

On the GeneCards, OMIM, and DisGeNET databases, “inflammation” was used as the key word in the search, and the results of the three databases were summarized and repeated targets were deleted. A total of 3184 disease targets were obtained. The compound targets were searched by SwissTargetPrediction. After drawing the Venn diagram and the intersection of “inflammation”-disease-related targets, ardisiacrispin B had 73 targets (Figure 4a), and its 26 metabolites had 164 targets (Figure 4b). The specific targets of ardisiacrispin B and its metabolites related to inflammation are shown in Tables S1 and S2.

The intersecting targets of ardisiacrispin B and its 26 metabolites related to disease were further analyzed for Kyoto Encyclopedia of Genes and Genomes (KEGG) and Gene Ontology (GO) enrichment (Figure 4c–f). The results revealed that the PI3K-AKT pathway may have an important role in the mechanism of action of ardisiacrispin B and its metabolites in exerting anti-inflammatory effects, and we subsequently validated the core targets related to the important pathway using LPS-stimulated RAW264.7 cells as a model.

### 2.3. Anti-Inflammatory Effect

**Figure 5:**
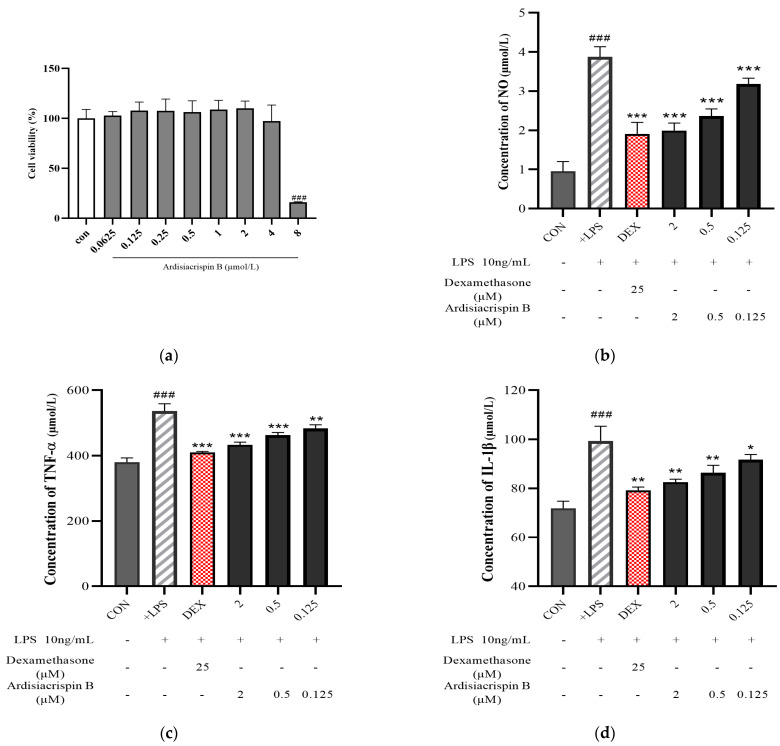
Effect of ardisiacrispin B on RAW264.7 (**a**) cell viability and on (**b**) NO, (**c**) TNF-α, and (**d**) IL-1β secretion in LPS-stimulated RAW264.7 cells. *N* = 5. Note: In cell-viability study, “con” means control cells, representing RAW264.7 cells without any treatment. In the measurement of inflammatory-factor secretion, “CON” represents RAW264.7 cells that were only treated with 2 mL DMEM medium containing 10% fetal bovine serum. The “+LPS” represents RAW264.7 cells that underwent treatment with 10 ng/mL LPS for 4 (TNF-α and IL-1β) or 24 (NO) hours to induce inflammation after being pre-treated with solvent (DMSO) for 24 h. The “DEX” refers to the use of cells pre-treated with 25 μM dexamethasone for 24 h prior to the induction of inflammation by LPS, which was used as a positive control in the experiment. The “2, 0.5, and 0.125 μM ardisiacrispin B” refers to the use of cells pre-treated with different concentrations of ardisiacrispin B for 24 h prior to the induction of inflammation by LPS, which were used as treatment cells in the experiment. vs. CON, ^###^ *p* < 0.001; vs. +LPS, *** *p* < 0.001, ** *p* < 0.01, * *p* < 0.05.

As shown in Figure 5a, there was no significant difference between the control cells (con) and ardisiacrispin B with different concentrations (0–4 μM). Thus, concentrations of ardisiacrispin B below 4 μM were selected for the subsequent experiments. To determine whether ardisiacrispin B has anti-inflammatory effects, the effects of ardisiacrispin B on NO, TNF-α, and IL-1β were assessed. Ardisiacrispin B dramatically inhibited the secretion of NO, TNF-α, and IL-1β, which were induced by LPS (Figure 5b–d). In the cells stimulated with LPS, the inflammatory cytokines increased significantly compared with the control cells, while in the treatment cells (2, 0.5, and 0.125 μM ardisiacrispin B), the inflammatory cytokines decreased significantly compared with the cells stimulated with LPS. Meanwhile, a significant concentration dependence was observed in this concentration range, indicating the remarkable anti-inflammatory effect of ardisiacrispin B.

### 2.4. Protein Expressions of AKT, P-AKT, PI3K, and P-PI3K

**Figure 6:**
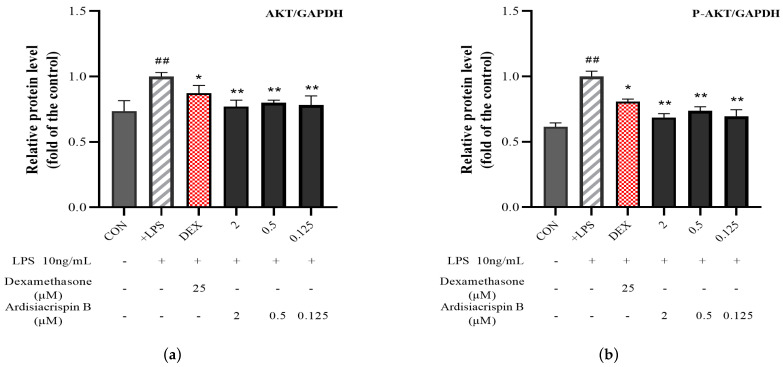

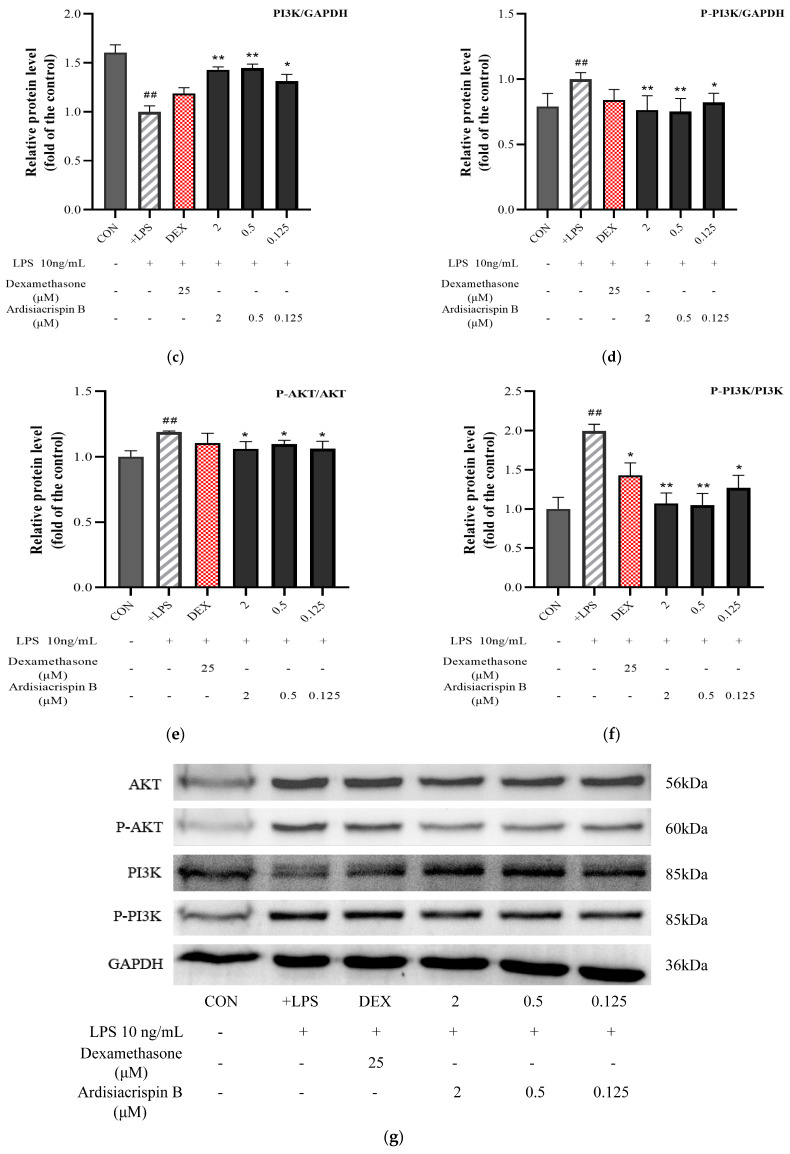
Protein expressions of (**a**) AKT, (**b**) P-AKT, (**c**) PI3K, (**d**) P-PI3K, (**e**) P-AKT/AKT, and (**f**) P-PI3K/PI3K after modeling and after administration of ardisiacrispin B in RAW264.7 cells, and (**g**) their Western blot protein bands. *N* = 3. Note: “CON” represents RAW264.7 cells that were only treated with 2 mL DMEM medium containing 10% fetal bovine serum. The “+LPS” represents RAW264.7 cells that underwent treatment with 10 ng/mL LPS for 24 h to induce inflammation after being incubated with solvent (DMSO) for 6 h. The “DEX” refers to the use of cells incubated with 25 μM dexamethasone for 6 h prior to the induction of inflammation by LPS, which was used as a positive control in the experiment. The “2, 0.5, and 0.125 μM ardisiacrispin B” refers to the use of cells incubated with different concentrations of ardisiacrispin B for 6 h prior to the induction of inflammation by LPS, which were used as treatment cells in the experiment. vs. CON, ^##^ *p* < 0.01; vs. +LPS, ** *p* < 0.01, * *p* < 0.05.

The specific data-processing steps in the Western blot experiment’s results were as follows. The gray value of each protein band was read by Image J 1.8.0 software. Next, the gray value of the target protein was divided by the gray value of the internal reference band, and the result was the relative protein content of the target band. Finally, the calculation results for each kind of protein are represented by p-protein/protein. The relative protein content of each cell was used for the statistical analysis, and the differences between the cells were compared. As shown in Figure 6, the protein expressions of AKT, P-AKT, PI3K, and P-PI3K showed significant changes in the treatment cells compared with those in the LPS-stimulated cells. Specifically, compared with the protein expression of the AKT in the LPS-stimulated cells, the protein expressions of the low-, middle-, and high-concentration-treatment cells decreased by 19.76%, 18.01%, and 20.91%, respectively. The protein expressions of the low-, middle-, and high-concentration-treatment cells dropped by 35.18%, 25.32%, and 30.03% in comparison to the protein expression of the P-AKT in the LPS-stimulated cells. The PI3K protein expressions in the low-, middle-, and high-concentration-treatment cells increased by 29.56%, 43.39%, and 42.17% compared to the LPS-stimulated cells. The protein expressions of the low-, middle-, and high-concentration-treatment cells decreased by 17.80%, 24.61%, and 23.54% in comparison to the protein expression of the P-PI3K in the LPS-stimulated cells. Compared with the control cells, the LPS-stimulated cells showed a significant increase in AKT-phosphorylation levels and a significant decrease in PI3K-phosphorylation levels (*p* < 0.01). Compared with the LPS-stimulated cells, the ardisiacrispin B showed a decrease in AKT-phosphorylation levels in cells along with an increase in PI3K-phosphorylation levels (*p* < 0.05). In short, all the concentrations of ardisiacrispin B showed efficacy in the four proteins.

## 3. Discussion

So far, scholars in China and abroad have isolated 103 compounds from *Ardisiae Crenatae Radix*, including 30 triterpenoids [22,23,24,25,26,27,28,29,30,31], two triterpenoid saponins [23,30,31], 12 coumarins [22,23,24,32,33], one sugar [32], 17 flavonoids [33], three quinones [22,34], three steroids [22,32,34], one sesquiterpene [35], two monoterpenes [35], 11 organic acids [23,33,35], and 21 others [35,36,37]. The results showed that the *Ardisiae Crenatae Radix* was mainly composed of triterpenoids, coumarins, and flavonoids. The research group established an LPS-stimulated RAW264.7 cell and investigated the changes in anti-the inflammatory activity of the *Ardisiae Crenatae Radix* extract before and after refining (refined by n-butanol—70% ethanol). The results showed that at the same crude drug concentration, the refined samples still significantly reduced the levels of NO, IL-1β, and TNF-α in the cell supernatant (*p* < 0.01). The acute-inflammation model of rat-paw swelling induced by carrageenan was established. The effects of the *Ardisiae Crenatae Radix* extract before and after refining on the degree and morphology of rat-paw swelling were compared. It was found that the refined *Ardisiae Crenatae Radix* extract still significantly reduced the degree of rat-paw swelling (*p* < 0.01). In the refined products, bengenin, 11-O-galloylbergenin, and ardisiacrispin B accounted for 80% of the total solid content [38]. This indicates that they may be active anti-inflammatory ingredients in the refined products of *Ardisiae Crenatae Radix*. Many reports have suggested that bengenin [16] and 11-O-galloylbergenin [17] have good anti-inflammatory effects, but the anti-inflammatory effect of ardisiacrispin B has not been reported. 

Ardisiacrispin B, under the chemical name sikramin A 3-O-α-L-rhamnopyranose-(1→2)-β-D-glucopyranose-(1→4)-[β-D-glucopyranose-(1→2)-]-α-L-arabinopyranoside, is found in several species of the *Ardisia* genus, including *Ardisia crenata* Sims, *Ardisia crispa* (Thunb.) A. DC., *Ardisia mamillata* Hance, and others. In preliminary research, it was found that its content in *Ardisia crenata* Sims is as high as 16.27%, and it is one of the main components of this herbal medicine [15]. Ardisiacrispin B has been reported in the literature to have uterotonic effects [39], to inhibit cancer cells, and to be cytotoxic [40], but its involvement in the inflammatory response has not been reported. The results of the study on the anti-acute-lung-injury mechanism of *Ardisiae Crenatae Radix* showed that the refined products of ardisiacrispin B and *Ardisiae Crenatae Radix* inhibited the inflammatory level of the acute-lung-injury model of rats to different degrees at 3, 5, 7, and 14 days. It is suggested that ardisiacrispin B may have the same anti-inflammatory effect as the refined products of Ardisiae Crenatae Radix [38]. Therefore, in this paper, we present the pharmacodynamic study of ardisiacrispin B in vitro, in which we found that ardisiacrispin B can indeed inhibit the LPS-induced secretion of NO, TNF-α, and IL-1β in RAW264.7 cells. This result is consistent with previous speculation.

In metabolic research, UHPLC coupled with high-resolution mass spectrometry can be used to provide complete information on the chemical composition of a sample, so the appropriate chromatographic and mass-spectrometry conditions can be used for better identification of components. In order to obtain well-separated chromatograms, several different mobile-phase systems, including methanol, acetonitrile, water, and different proportions of formic acid, were examined in the metabolite-analysis study. It was found that 0.1% formic acid in water and 0.1% formic acid in methanol provided lower background noise, better peak shapes, and higher intensities in both positive and negative modes, which may be related to the chemical nature of ardisiacrispin B terpenoids. According to the working principle of Orbitrap, it is possible to scan for information about compounds with intensities in the TOP-N range. In order to obtain as much information about the compounds as possible, the liquid-phase conditions require that the peaks are separated as much as possible and uniformly distributed in all segments of the chromatogram, so the final optimization of the existing chromatographic mass-spectrometry conditions was obtained.

Pentacyclic triterpenoids are generally more lipid-soluble due to their polycyclic alkane structure, and the amount of drug absorbed into the blood circulation after oral administration is low, resulting in low bioavailability, which severely limits the clinical use of such compounds [41,42]. Therefore, the clarification of the in vivo metabolic profiles of pentacyclic triterpenoids after oral absorption is important for improving bioavailability and promoting the use of this class of compounds in the future [43]. Most of the pentacyclic triterpenoids in nature combine with sugar groups to form glycosides in the form of saponins. Most of the pentacyclic triterpenoid saponins are mainly converted into glycosides by various enzymes and bacteria to produce biological activities, and they are absorbed in the intestinal tract and exert pharmacological effects in the form of glycosides [44,45,46].

Most of the ardisiacrispin B metabolites identified in this study were also converted to sikramin A before further metabolism. Ardisiacrispin B undergoes a number of one-phase and two-phase metabolic processes, such as oxidation and binding reactions, and it is most commonly converted to water-soluble compounds for elimination from the body. The postulated metabolic process is essentially consistent with the metabolism of pentacyclic triterpene saponins in vivo [44]. Numerous findings have shown that saponins are changed into deglycosylated products in vivo after conversion by intestinal action, thus exerting or enhancing pharmacological effects. The original ginsenoside is transformed by the intestinal flora, hydrolyzed by glycosidase, and deglycosylated to produce secondary ginsenosides or glycosides, such as ginsenosides Rh2, CK, and PPD. The above metabolites inhibit epithelial–mesenchymal transition, glycolysis, the activation of endoplasmic reticulum stress, and other mechanisms, and ultimately exert stronger anti-tumor effects than prototype ginsenosides [47,48,49,50].

Based on the analysis of plasma metabolites, in this study, we conducted a network-pharmacology analysis on the effects of the prototype of ardisicrispin B and of its plasma metabolites on inflammation. The predicted common signaling pathway can not only reflect the anti-inflammatory pathway of ardisicrispin B itself, but can also take into account the potential anti-inflammatory characteristics of its metabolites. It may be able to more comprehensively map the role of ardisicrispin B. The enrichment-analysis results of the two parts showed that the PI3K-AKT pathway was located in the top 10, indicating that the prototype and metabolites of ardisicrispin B may exert anti-inflammatory effects by affecting this pathway.

The PI3K/AKT signaling pathway is one of the important regulatory pathways in cells. It participates in the regulation of various biological behaviors such as cell proliferation, apoptosis, and invasion in malignant tumors by affecting the abnormal expression of related genes on its signaling pathway. The PI3K group is a group of plasma-membrane-associated lipid kinases that can be divided into three categories (class I, II, and III) based on their structure, substrate specificity, and reaction mechanism. Among these categories, class I PI3K has been the most extensively studied [51]. Class I PI3K is the main structure of PI3K and is a heterodimer composed of the regulatory subunit p85 and the catalytic subunit p110. The amino terminus of p85 contains an Src homology 3 (SH3) domain and two proline-rich regions, whereas the basal terminus contains two SH2 domains and a noncoding region that binds p110. In most cases, p85 is inhibited, and class I PI3K has little kinase activity. [52]. In addition, there are four subtypes of class I PI3K, including class IA (PI3Kα, PI3Kβ, PI3Kδ) and class IB (PI3Kγ). The former class is activated by growth-factor-receptor tyrosine kinases, and the latter by G-protein-coupled receptors [53]. Class II PI3K has only a catalytic subunit and no regulatory subunits, and it is mainly involved in the production of phosphatidylinositol 3-phosphate and phosphatidylinositol 3,4-bisphosphate. Only one member of class III PI3K, Vsp34, has been found so far, and it is mainly involved in autophagy [54]. Furthermore, AKT is a key target protein downstream of the PI3K signaling pathway and is a serine/threonine protein kinase. It has three different isoforms: AKT1 (PKBα), AKT2 (PKBβ), and AKT3 (PKBγ). These three AKT isoforms are mainly composed of the Pleckstrin homology (PH) domain, kinase catalytic structure, regulatory structure, and composition [55]. Furthermore, AKT1 is widely expressed in most tissues, AKT2 is mainly expressed in insulin-sensitive tissues, and AKT3 is specifically expressed in brain and testicular tissues [52]. The analyses of the plasma metabolites and the network pharmacology revealed that ardisiacrispin B and its army of metabolites may exert anti-inflammatory effects by modulating the PI3K-AKT pathway. The pharmacodynamic and protein-expression experiments revealed that the ardisiacrispin B reduced AKT-, P-AKT-, PI3K-, and P-PI3K-protein expression in LPS-induced RAW264.7. In the growth-factor superfamily, PI3K is an important signal-transduction molecule, and AKT is a serine/threonine kinase, which is a key mediator of PI3K-mediated signal transduction [56,57]. The phosphorylation of its serine and threonine residues activates AKT with the help of PI3K-dependent kinase (PDK). The PI3K-AKT signaling pathway plays a key role in various cellular processes, including cell survival, growth, and proliferation [58,59]. Activated AKT can continue to act on downstream target proteins, such as mTOR, NF-κB, FoxO3a, GSK3, p21, p27, S6K1, and Bcl-2, to produce cellular responses. It plays a key role in multiple processes, such as cell proliferation, autophagy, apoptosis, the cell cycle, cell senescence, and the inflammatory response [60].

In the pre-experiment, the secretion levels of TNF-α, IL-1β, and NO at 4, 8, 12, 24, and 48 h after LPS stimulation were investigated. The results showed that there was a significant difference in the secretion of NO after LPS-stimulated cells for 24 h compared with the control cells. After 4 h of stimulation, there was a significant difference in the secretion of TNF-α and IL-1β between the two cells. Therefore, there are two different kinds of LPS-stimulation time. When LPS stimulates the body, it initiates a multi-step signaling-cascade reaction of various cells in the tissue, leading to the production of various inflammatory mediators, including pro-inflammatory factors, chemokines, adhesion molecules, and others [61]. Because TNF-α and IL-1β are the pro-inflammatory cytokines that are released the earliest during the LPS-mediated inflammatory response, TNF-α is the initiating factor, while IL-1β plays a synergistic role. At the same time, endothelial cells can be directly activated to regulate inflammatory responses and damage tissues and organs by producing NO [62]. Therefore, NO, TNF-α, and IL-1β were selected as pharmacodynamic indicators for examination. In addition, dexamethasone can promote the synthesis of anti-inflammatory media and inhibit the release of inflammatory mediators by inflammatory cells. Furthermore, dexamethasone is a first-line clinical medication with a fast onset and good therapeutic effects on inflammatory reactions caused by various factors [63]. A low dose of dexamethasone can significantly improve the release of inflammatory mediators, thereby improving the incidence rate and mortality [64]. Therefore, dexamethasone is often selected as a positive-control drug in research on inflammation-related diseases.

## 4. Materials and Methods

### 4.1. Chemicals and Reagents

The ardisiacrispin B used in this study was extracted and isolated from medicinal materials of *Ardisiae Crenatae Radix*, which was prepared in the Guizhou Provincial Key Laboratory of Pharmaceutics with purity > 98%, batch number 20211204. The specific identification information can be found in Figure S2.

The medicinal materials of *Ardisiae Crenatae Radix* were purchased from the Wandongqiao Pharmaceutical Market (Guiyang, China) and identified as the dry roots of *Ardisia crenata* Sims, a plant of the *Ardisia* genus, *Primulaceae* family, by Associate Professor Chunhua Liu, School of Pharmacy, Guizhou Medical University.

Lipopolysaccharide (LPS, lot. 0000153963) was purchased from Sigma-Aldrich Co., Ltd. in City of Saint Louis, MO, USA. Dexamethasone (lot. 110K053), penicillin–streptomycin mixture (lot. 20210119), PBS buffer (lot. 8121682), dimethyl sulfoxide (DMSO, lot. 401F0322), heparin sodium (lot. 125P0216), glycine (lot. 923W062), Tris (lot. 117C074), SDS (lot. 526K031), tween-20 (lot. 223Y013), rainbow 180 spectral protein marker (lot. 104K022), bovine serum albumin V (lot. 120O059), 5x-protein-loading buffer solution (lot. 20220919), and BCA protein-concentration-assay kit (lot. 20210901) were obtained from Beijing Solarbio^®^ Life Sciences Co., Ltd. in Beijing, China. Fetal calf serum (FBS, 2375386CP) and DMEM high-glucose medium (lot. 8121599) were purchased from Gbico^TM^ Co., Ltd. in Grand Island, NY, USA. The RIPA lysate (lot. MA0151-Jul-13H) and Feiket ultrasensitive ECL luminescent solution (lot. MA0186-Dec-12H) were obtained from Dalian Meilunbio^®^ Biotechnology Co., Ltd. in Dalian, China. The PVDF membrane (lot. R1SB05193C) was purchased from Millipore^®^ in Billerica, MA, USA. The NO assay kit (lot. 20220261) was obtained from Nanjing Jiancheng Bioengineering Institute (Nanjing, China). A rapid-preparation kit for denatured acrylamide gel was purchased from BBI Life Sciences Corporation (Shanghai, China). The available ELISA kits were obtained from Shanghai Zcibio Technology Co., Ltd. (Shanghai, China), and were used to determine the levels of TNF-*α* and IL-1*ß*. All other chemicals used in this study were of analytical grade.

### 4.2. Animals and Cells

Animals: Twenty SPF-grade male Sprague Dawley rats weighing 200 ± 20 g (8–10 weeks old) were supplied by Changsha Tianqin Biotechnology Co., Ltd. with license no. SCXK (Xiang) 2019-0014. Once the rats entered the animal house, they were managed in packs of five per cage, fed with standard chow, and provided with fresh, purified water that was replaced daily. The animal house was well lit with a 12-hour day/night cycle, well ventilated, and maintained at a room temperature of 22 ± 2 °C and a relative humidity of 15–60%, and it required regular disinfection. Prior to the experiment, rats were adaptively reared in the laboratory for 7 days. All animal trials were approved by the Animal Ethics Committee of Guizhou Medical University (1603125). These animals were randomly divided into a blank control group and an ardisiacrispin-B-administrated group. The blank control group was gavaged water, and the ardisiacrispin-B-administered group was gavaged with 50 mg/kg of ardisiacrispin B aqueous solution (concentration of 6 mg/mL), twice a day, for 3 consecutive days.

Cell line: RAW 264.7 (mouse mononuclear macrophage cells) was purchased from Wuhan Punosai Biotechnology Co., Ltd. The RAW264.7 were cultured at 37 °C in a 5% CO_2_ incubator. When the cell density of the culture bottle reached about 80%, the old culture medium was sucked out, and the PBS buffer was added for wetting. Next, the buffer solution was poured out, and 4 mL of culture medium was added. The cells at the bottom of the culture bottle were blown and beaten until they were completely blown down. The cell solution was passaged in a 1:1 ratio and cultured in a 5% CO_2_ incubator at 37 °C. The RAW264.7 cells in stable growth conditions and in logarithmic phase were taken and laid in 96-well plates with a density of 2 × 10^5^ cells/mL, and 100 μL/hole. In addition, PBS was added to peripheral edge holes to prevent the edge effect. The inoculated cells were cultured overnight in a 5% CO_2_ incubator at 37 °C. When more than 80% of the cells adhere to the wall completely, it can be used.

### 4.3. Isolation of Ardisiacrispin B

The isolation of pure ardisiacrispin B products was carried out by the method in the literature for subsequent experiments [23,65]. Four kilograms of *Ardisiae Crenatae Radix* were extracted twice with 80% ethanol. The extract was filtered, and ethanol was recovered with no taste of alcohol. The residue was dissolved in water to 2000 mL and extracted three times with the same amount of petroleum ether (2000 mL each time). After the water layers were combined, the same amount of ethyl acetate (2100 mL each time) was added for extraction three times. After merging the water layer again, the same amount of n-butanol (2500 mL each time) was added for extraction three times, the n-butanol layer merged, the n-butanol recovered under reduced pressure, and a total of 885 g of partial extract of n-butanol from *Ardisiae Crenatae Radix* was obtained (partial n-butanol extracts yielded about 22.13%).

The partial n-butanol extract was divided into ten parts, each of which was dissolved in 200mL water, adsorbed with 315 g pretreated D101 macroporous resin, and then eluted with water, 30% ethanol, 50% ethanol, 70% ethanol, and 90% ethanol, respectively. The 70% ethanol elution was combined, and 207 g of ethanol extract of *Ardisiae Crenatae Radix* was recovered under reduced pressure (70% ethanol extracts yielded about 5.18%).

The column was loaded by the dry method, and the silica gel with uniform sample mixing was placed on the surface of the loaded silica-gel column, eluted with dichloromethane ethyl acetate methanol (3:1:0.5), and collected in sections. Each section was sampled on a silica-gel GF254 prefabricated plate, developed with dichloromethane ethyl acetate methanol (3:1:0.5), and colored with sulfuric-acid-ethanol solution, combined with 12–22 parts, and solvent was recovered to obtain sample A (39.01 g, yielding about 0.98%). Sample A was loaded onto the column five times, dissolved in 20 mL of 60% methanol each time, and purified by methanol-water-column chromatography on a reversed-phase ODS silica gel column. Sample A was dissolved in 60% methanol, subjected to a reverse phase ODS silica gel column, purified by methanol-water-column chromatography (from 60% to 70%, gradient of 5%, 400 mL each), and collected in sections. Each segment was spotted on a C18 reverse-octadecylated thin-layer silica-gel plate, developed with 60% methanol, colored with sulfuric-acid-ethanol solution, and merged with 38–45 parts, and the solvent was recovered to obtain sample B. After recrystallization of sample B, ardisicrispin B was obtained, totaling 7.99 g (the ardisicrispin B yield was about 0.2%).

### 4.4. Plasma-Metabolite Research

#### 4.4.1. Preparation of Plasma-Containing Drugs

Metabolite research was set up as a blank control group and an ardisiacrispin-B-administered group (*N* = 10). The rats were fasted without water for 12 h before the experiment. At the beginning of the experiment, the blank control group received water, and the administered group was gavaged ardisiacrispin B (50 mg/kg rat *b.w.*, 6 mg/mL; solvent was water). Each group of rats was administered their respective treatment twice a day, with a 12-hour interval between the two doses. After 3 days of continuous administration, 0.5 mL of blood was taken from the rats via the tail vein after 10, 30, 60, 90, 120, 150, and 240 min. The blood samples were incubated in a water bath at 37 °C until a yellow liquid (plasma) precipitated from the upper layer, followed by centrifugation at 5000 rpm and 4 °C for 10 min. Samples from the same group of rats at each time point were mixed, and 1 mL was taken to obtain mixed plasma samples from the control and administered groups.

In the mixed plasma, 400 μL of 0.1% formic acid and 4 mL of methanol were added to precipitate the proteins, and then vortexed for 3 min, sonicated for 10 min, and centrifuged at 8000 rpm at 4 °C for 10 min to obtain the supernatant. The supernatant was transferred and dried at 37 °C under nitrogen. Furthermore, 200 μL of 50% methanol was added to the residue, and then vortexed for 3 min, sonicated for 10 min, and centrifuged for 10 min at 12,000 rpm, and the supernatant was obtained and injected into the instrument.

#### 4.4.2. Instrument Conditions

The instrument and chromatographic column models were as follows: Vanquish Horizon ultra-high-performance liquid chromatography (UHPLC, Thermo Fisher Scientific Corp., Waltham, MA, USA); Q Exactive Orbitrap Plus MS (Thermo Fisher Scientific Corp., Waltham, MA, USA); electronic spray ion source (ESI); Hypersil GOLD C_18_ column (100 mm × 2.1 mm, 1.9 µm, Thermo Fisher Scientific Corp., Waltham, MA, USA).

Scan mode was full-scan MS/dd-MS^2^. The scan range was from *m*/*z*100 to 1500. Spray voltages were 3.0 kV for positive mode and 2.5 kV for negative mode. Resolutions were 70,000 for MS^1^ and 17,500 for MS^2^. Both sheath gas and AUX gas were N_2_, and their flow rates were 40 arb and 10 arb, respectively. The capillary temperature was 320 °C. The S-Lens RF level was 50. In total, 0.1% formic methanol (A) and 0.1% formic water (B) were used for the mobile phase at a flow rate 0.3 mL/min. Column temperature was 40 °C. Sample volume was 3 µL. With gradient elution, the elution procedure was 0–3 min, 2% A, 3–6 min, 2–35% A, 6–12 min, 35–50% A, 12–15 min, 50–75% A, 15–20 min, 75–85% A, 20–25 min, 85–98% A.

### 4.5. Network Pharmacology

The structures of ardisiacrispin B and all its metabolites were converted into “.sdf” format and uploaded to the SwissTargetPrediction database (www.swisstargetprediction.ch, accessed on 13 September 2023). The predicted targets of the prototype and each metabolite were obtained, and probability >0 was used as the inclusion criterion. Finally, the protein targets of each component were standardized in the UniProt protein-resource database.

Relevant disease targets were searched using the keyword “inflammation” in the GeneCards (www.genecards.org, accessed on 13 September 2023, relevance score > 1), OMIM (www.omim.org, accessed on 13 September 2023), and DisGeNET (www.disgenet.org, accessed on 13 September 2023) databases. Finally, the results from the three databases were combined to obtain disease-associated targets after removing duplicate values.

The intersection of ardisiacrispin B and inflammation-predictive targets was obtained by “Draw Venn Diagram,” an online program in R. The cellular components, molecular functions, biological processes, and signaling pathways of the intersecting genes were enriched and analyzed based on R to infer the signaling pathways.

### 4.6. Anti-Inflammatory Mechanisms

#### 4.6.1. Determination of Cell Viability

Ardisiacrispin B was dissolved in DMSO and precisely prepared into a stock solution with a concentration of 100 mM, which was stored at −20 °C for future use. Cell viability was monitored with the CCK8 kit (CCK8, GlpBio Co., Ltd., USA) following the producers’ suggestions. Logarithmic growth-phase cells were taken and spread in 96-well plates at 100 μL per well, 2 × 10^5^ cells/mL. Subsequently, 100 μL of ardisiacrispin B was added at concentrations of 0.0625, 0.125, 0.25, 0.5, 1, 2, 4, and 8 μM, respectively (diluted with DMEM medium containing 10% serum). In addition, there was a control cell (denoted as “con,” containing cells and without drugs), and all experimental cells had five re-wells. After 24 h of incubation in the incubator, the old medium was sucked out, and the PBS was rinsed twice. After incubation, 10% CCK8 solution was added to each well and incubated at 37 °C for 1.5 h. Subsequently, the absorbance was measured at 450 nm using a microplate reader. Cell viability (%) = (A_treatment_ − A_blank_)/(A_control_ − A_blank_) × 100%.

#### 4.6.2. Detection of the Contents of NO, TNF-α, and IL-1β in Cells

Six groups were set up for the experiment, namely control cells (CON), LPS-stimulated cells (10 ng/mL, +LPS), dexamethasone-treated cells (DEX), and ardisiacrispin B-treated cells with three concentrations (“2,” “0.5,” and “0.125”). In total, 100 μL of DMEM medium containing 10% fetal bovine serum was added into each well of the control cells. The “+LPS” cells were pre-treated with solvent (DMSO) for 24 h prior to the induction of inflammation by LPS. In total, 100 μL of 0.125, 0.5, and 2 μM ardisiacrispin B, as well as 25 μM dexamethasone, were added to the three concentrations of ardisiacrispin-B-treated cells and dexamethasone-treated cells, respectively. The cells were treated with the drug or DMEM medium containing 10% fetal bovine serum for 24 h, and the cell supernatant was discarded. In addition to the control cells, 100 μL of serum-free LPS (10 ng/mL) stimulated the cells for 4 h and 24 h, respectively.

After 4 h, the supernatant of the cells was taken and operated according to the instructions in the ELISA kit. The absorbance value was measured at 450 nm by the microplate reader and then substituted into the standard curve to calculate the contents of TNF-α and IL-1β. After 24 h, the cell supernatant was taken and operated according to the instructions in the NO kit. The absorbance value was measured at 550 nm by the microplate reader and substituted into the standard curve to calculate the content of NO.

#### 4.6.3. Western Blot

The RAW264.7 cells in the logarithmic phase of growth were selected and spread in 6-well plates at 2 mL/well, 1 × 10^6^ cells/mL. The inoculated cells were cultured in a 5% CO_2_ incubator at 37 °C for 6 h. When the cells adhered completely, the 6-well plates were taken out, and the cells were divided into control cells (CON), LPS-stimulated cells (10 ng/mL, +LPS), dexamethasone-treated cells (DEX), and ardisiacrispin B-treated cells, with three concentrations (“2”, “0.5”, and “0.125”). In control cells, 2 mL of DMEM medium containing 10% fetal bovine serum was added to each well. In dexamethasone-treated cells, 2 mL of DMEM medium containing dexamethasone (final concentration is 25 μM) was added to each well. In low-, middle-, and high-concentration-treatment cells, 2 mL of DMEM medium containing different concentrations of ardisiacrispin B was added to each well (final concentrations are 0.125, 0.5, and 2 μM). For LPS-stimulated cells, 2 mL of DMEM medium containing DMSO was added to each well. After adding drugs or culture medium, the cells were incubated again for 6 h. Except for control cells, 2 mL of serum-free LPS (10 ng/mL) was added to each well for 24 h.

Next, 250 μL of cell lysate containing 1% PMSF was placed on ice for full lysis and centrifuged to obtain cell supernatant. The protein concentration was determined by the BCA kit, and all sample protein concentrations were diluted to the same level (5 mg/mL). An appropriate amount of 1x loading buffer was added to the protein sample, boiled after vortex mixing, taken out and cooled, and stored at −20 °C for future use. Subsequently, the protein samples were separated on 10% SDS polyacrylamide gels and electro-transferred to a polyvinylidene fluoride membrane. The membranes were blocked with 5% BSA for 1 h at 30 °C and then incubated with the following primary antibodies overnight at 4 °C: GAPDH (1:10,000, mouse monoclonal, Proteintech Group, Inc., Chicago, IL, USA, 10029187), AKT (1:2000, rabbit polyclonal, Proteintech Group, Inc., Chicago, IL, USA, 00114013), P-AKT (1:2000, mouse monoclonal, Proteintech Group, Inc., Chicago, IL, USA, 10022023), PI3K (1:5000, mouse monoclonal, Proteintech Group, Inc., Chicago, IL, USA, 10005702), and P-PI3K (1:500, rabbit polyclonal, Zenbio Science, Chengdu, China, L28NO11). On the next day, the membranes were incubated with a 1:10,000 dilution of the secondary antibody for 2 h at room temperature. The gel was placed in the imaging system for photographing.

### 4.7. Statistical Analysis

All numerical data were expressed as mean ± sd. One-way analysis of variance (ANOVA) was used to analyze protein expressions in RAW264.7 cells, followed by post hoc tests using least significant difference (LSD). An independent-samples t test was used for the uptake and transport assays, and a difference of *p* < 0.05 was considered statistically significant. The rank-sum test was used for variance non-homogeneity. All the statistical analyses were performed with the Statistical Package for the Social Sciences, version 13.0 (SPSS, Chicago, IL, USA).

## 5. Conclusions

Overall, ardisiacrispin B is mainly converted to water-soluble components in vivo through multiple metabolic processes, such as oxidation, dehydration, glucoside conjugation, and others. Furthermore, it reduces the production of LPS-induced pro-inflammatory media, such as NO, TNF-α, and IL-1β, in RAW264.7 cells and exerts a block on the activation of the PI3K-AKT signaling pathway by inhibiting the phosphorylation of PI3K and AKT. This study fills the gaps in the knowledge on the in vivo metabolic process of ardisiacrispin B and explores its anti-inflammatory mechanism, providing an experimental basis for the development and utilization of pentacyclic triterpenoids.

## Figures and Tables

**Table 1:** The mass-spectrometry information for specific metabolites of ardisiacrispin B.

Name	Formula	RT	MS ^1^	ppm	MS ^2^	Area	Metabolic Type
Ardisiac rispin B (M)	C_53_H_86_O_22_	16.49	[M−H]^−^ 1073.5550	1.21	119.0323, 143.0320, 159.0268, 161.0420, 205.0695, 893.4904,911.4976, 927.4962, 971.5221	1.92 × 10^5^	Prototype
M1	C_6_H_12_O_6_	0.91	[M+Na]^+^ 203.0516	−4.92	68.0204, 85.5360, 120.4583, 134.0188, 143.0314	2.68 × 10^7^	Oxidation
M2	C_6_H_12_O_7_	0.94	[M−H]^−^ 195.0501	−4.61	75.0075, 129.0175, 160.8396	6.43 × 10^6^	Oxidation, oxidation
M3	C_12_H_22_O_11_	1.04	[M+Na]^+^ 365.1044	−2.74	185.0415, 203.0537	5.78 × 10^5^	Oxidation, glucoside conjugation
M4	C_6_H_10_O_10_S	1.89	[M+H]^+^ 275.0077	3.64	156.9802, 174.9911, 193.0011, 256.9966	7.06 × 10^6^	Oxidation, sulfation
M5	C_6_H_12_O_9_S	2.04	[M+H]^+^ 261.0283	3.45	138.9711, 146.9967, 156.9816, 174.9911, 193.0011, 235.0140	2.98 × 10^6^	Oxidation, sulfation
M6	C_6_H_10_O_2_	2.51	[M+NH4]^+^ 132.1015	−3.03	69.0703, 86.0972, 104.9639	3.51 × 10^7^	Dehydration, nitro reduction
M7	C_12_H_20_O_7_	3.05	[M+NH4]^+^ 294.1538	−3.06	97.0292, 127.0338, 132.1025, 230.1390, 258.1338, 276.1437	1.22 × 10^6^	Dehydration, nitro reduction
M8	C_6_H_10_O_5_	6.68	[M+FA−H]^−^ 207.0514	1.93	96.9587, 127.8675, 159.8575, 162.8349	1.71 × 10^5^	Dealkylation
M9	C_6_H_10_O_4_	6.86	[M+Na]^+^ 169.0475	2.37	56.9656, 84.9605, 108.9596	1.35 × 10^5^	Dehydration
M10	C_11_H_20_N_2_O_5_	7.62	[M+H]^+^ 261.1452	3.06	86.0971, 132.1014, 198.1128, 244.1171	6.36 × 10^6^	Dehydration, ornithine conjugation
M11	C_8_H_15_NO_4_	8.15	[M−H]^−^ 188.0922	−3.19	116.0705, 141.8659, 159.8768	7.66 × 10^5^	Nitro reduction, Glycine conjugation
M12	C_55_H_84_O_22_	16.55	[M+H]^+^ 1097.5559	2.92	275.0751, 421.1329, 643.2072, 951.4934	1.84 × 10^6^	Dehydration, acetylation
M13	C_30_H_48_O_4_	18.19	[M+Na]^+^ 495.3459	3.03	80.9486, 184.0734, 495.3459	9.16 × 10^5^	Dealkylation
M14	C_30_H_44_O_2_	18.20	[M+H]^+^ 437.3426	2.74	95.0862, 107.0856, 119.0856, 407.3309, 419.3309	1.51 × 10^5^	Dehydration, dehydration
M15	C_30_H_46_O_3_	18.20	[M+H]^+^ 455.3540	4.39	95.0861, 119.0854, 145.1015, 187.1483, 437.3434	1.47 × 10^5^	Dehydration
M16	C_12_H_20_O_12_	18.56	[M+Na]^+^ 379.0838	−2.11	149.0223, 222.9697, 253.0187, 266.9596, 365.1435	4.16 × 10^5^	Glucuronide conjugation
M17	C_35_H_58_N_2_O_6_	20.08	[M−H+HAc]^−^ 661.4452	2.87	179.1073, 230.0919, 262.0817, 301.2181, 319.2280, 341.2104	8.65 × 10^6^	Hydration, ornithine conjugation
M18	C_22_H_42_O_6_	22.90	[M+Na]^+^ 425.2887	3.29	90.9772, 220.9344, 288.9230	6.03 × 10^6^	Palmitoyl conjugation
M19	C_22_H_44_O_6_	23.06	[M+Na]^+^ 427.3011	−4.45	67.3221, 80.9490, 164.9196, 264.2387	5.31 × 10^5^	Palmitoyl conjugation, reduction
M20	C_47_H_74_O_16_	23.09	[M+H]^+^ 895.5073	2.57	83.0501, 111.0449, 129.0567, 215.1252, 583.2816, 783.3730	5.75 × 10^5^	dehydration, reduction
M21	C_32_H_47_NO_4_	23.13	[M+H]^+^ 510.3557	−3.92	104.1071, 125.0001, 184.0730	5.53 × 10^6^	Dehydration, glycine conjugation
		23.85	[M−H] 508.3423	−1.77	78.9579, 168.0417, 224.0686, 283.2650	1.68 × 10^7^	Dehydration, glycine conjugation
M22	C_22_H_40_O_5_	23.98	[M+Na]^+^ 407.2785	4.42	67.1376, 96.6027, 113.1913, 142.2898, 182.0834	9.91 × 10^5^	Palmitoyl conjugation, dehydration
M23	C_30_H_46_O_3_	26.00	[M+H]^+^ 455.3522	0.44	86.0970, 90.9773, 164.9203, 187.0367, 315.3575, 443.2406	1.02 × 10^6^	Dehydration
M24	C_30_H_44_O_3_	26.25	[M+H]^+^ 453.3344	−4.19	187.0367, 205.0476	1.26 × 10^6^	Dehydration
M25	C_31_H_48_O_3_	26.56	[M+H]^+^ 469.3656	−4.26	83.4855, 222.5850, 454.3398	1.51 × 10^7^	Dehydration, methylation
M26	C_31_H_48_O_4_	26.99	[M+H]^+^ 485.3618	−1.44	219.0620, 336.7990, 381.7993, 467.3460	2.90 × 10^5^	Desaturation, methylation

Note: The structures of ardisiacrispin B and its predicted metabolites in this table are shown in Figure 3. MS ^1^ represents primary mass spectrometry information and MS ^2^ represents secondary mass spectrometry information.

## Data Availability

The data used to support the findings of this study are available from the corresponding author upon request.

## Supplementary Materials

The following supporting information can be downloaded at: https://www.mdpi.com/article/10.3390/ijms242317059/s1.
