# Supplementary material for: Metabolites-Based Network Pharmacology to Preliminarily Verify In Vitro Anti-Inflammatory Effect of Ardisiacrispin B
Source: Int J Mol Sci. 2023 Dec 2;24(23):17059. doi: 10.3390/ijms242317059 (PMC10707123; doi:10.3390/ijms242317059)
Supplement: Supplementary file 1 [file ijms-24-17059-s001.zip › ijms-2686684-supplementary.pdf]

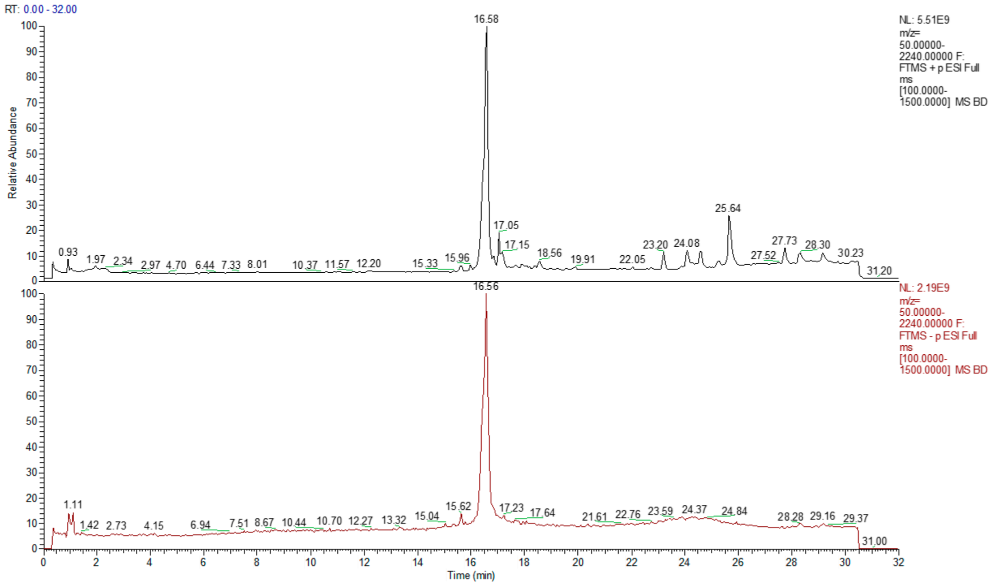
RT: 0.00 - 32.00
NL: 5.51E9 m/z= 50.00000-2240.00000 F: FTMS + p ESI Full ms [100.0000-1500.0000] MS BD
NL: 2.19E9 m/z= 50.00000-2240.00000 F: FTMS - p ESI Full ms [100.0000-1500.0000] MS BD
Relative Abundance
Time (min)

(**a**)

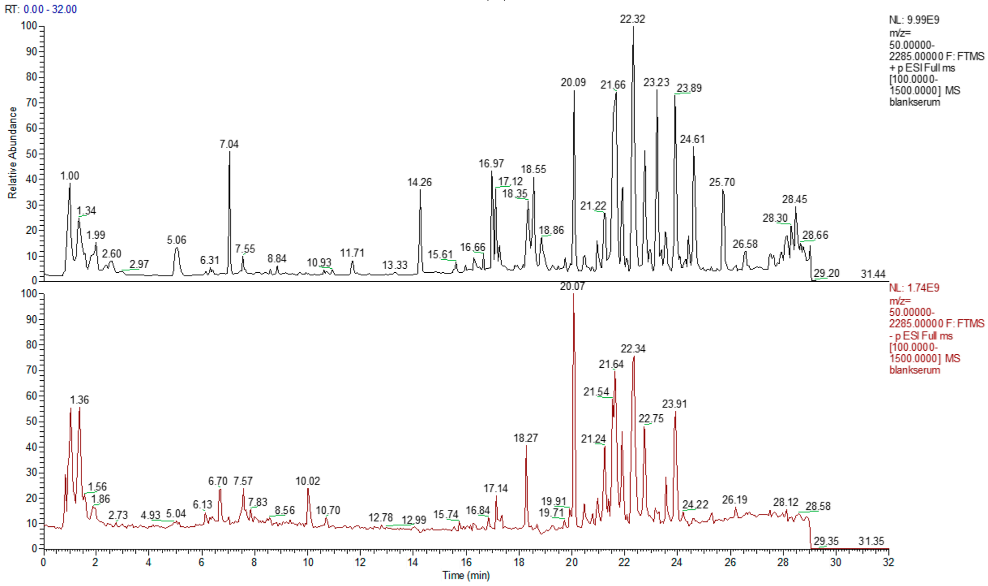
RT: 0.00 - 32.00
NL: 9.99E9 m/z= 50.00000-2285.00000 F: FTMS + p ESI Full ms [100.0000-1500.0000] MS blankserum
NL: 1.74E9 m/z= 50.00000-2285.00000 F: FTMS - p ESI Full ms [100.0000-1500.0000] MS blankserum
Relative Abundance
Time (min)

(**b**)

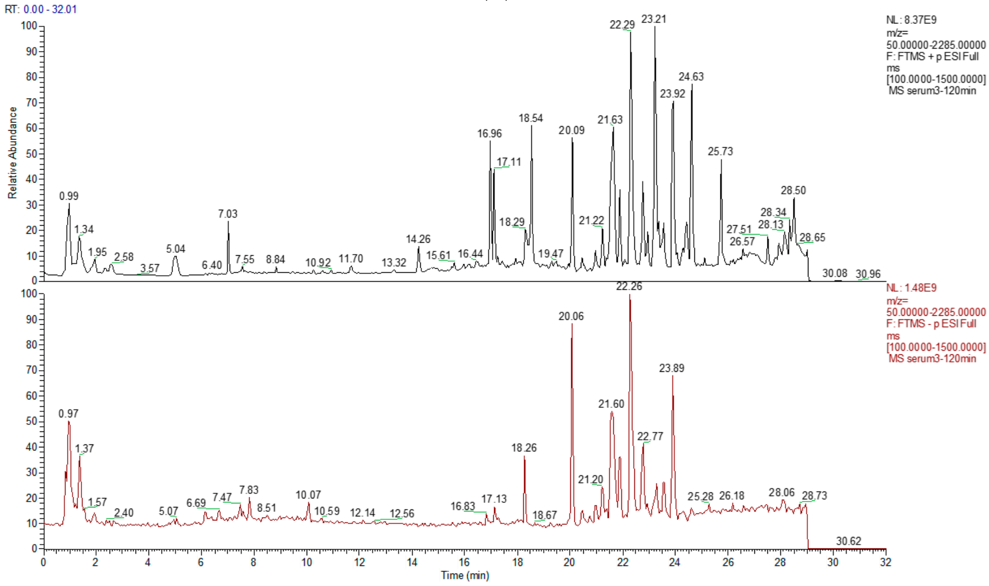
RT: 0.00 - 32.01
NL: 8.37E9 m/z= 50.00000-2285.00000 F: FTMS + p ESI Full ms [100.0000-1500.0000] MS serum3-120min
NL: 1.48E9 m/z= 50.00000-2285.00000 F: FTMS - p ESI Full ms [100.0000-1500.0000] MS serum3-120min
Relative Abundance
Time (min)

(**c**)

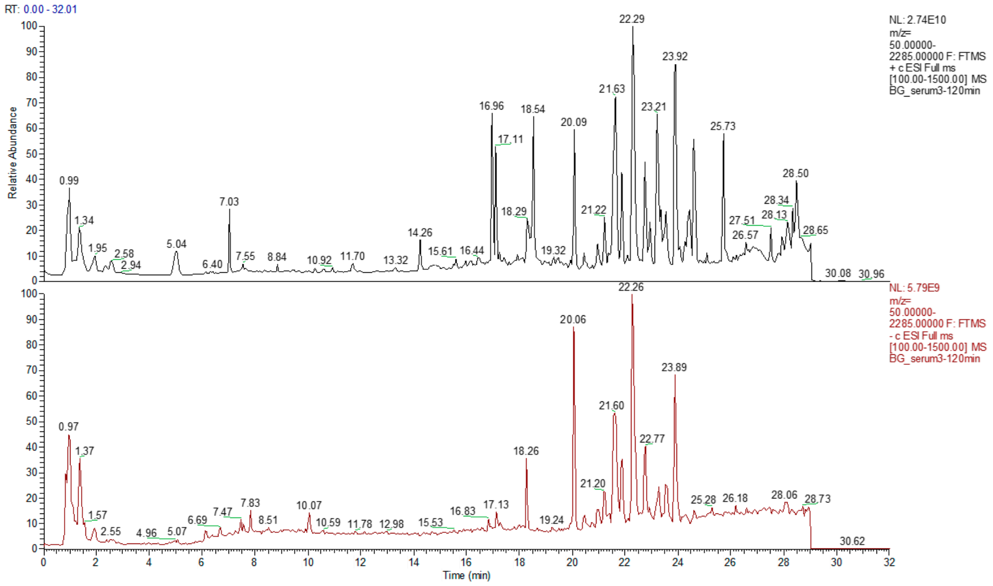

(**d**)

Figure s1. The total ion chromatograms of (**a**) ardisiacrispin B, (**b**) blank plasma, (**c**) drug-contained plasma, and (**d**) background subtracts.

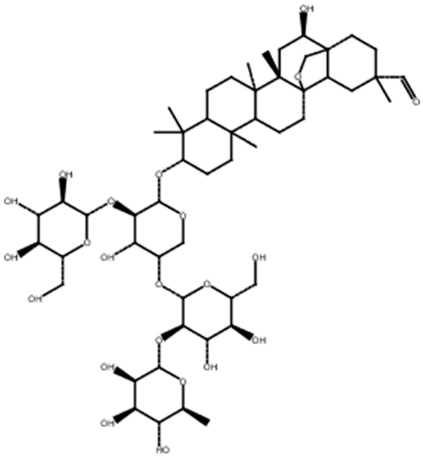

(**a**)

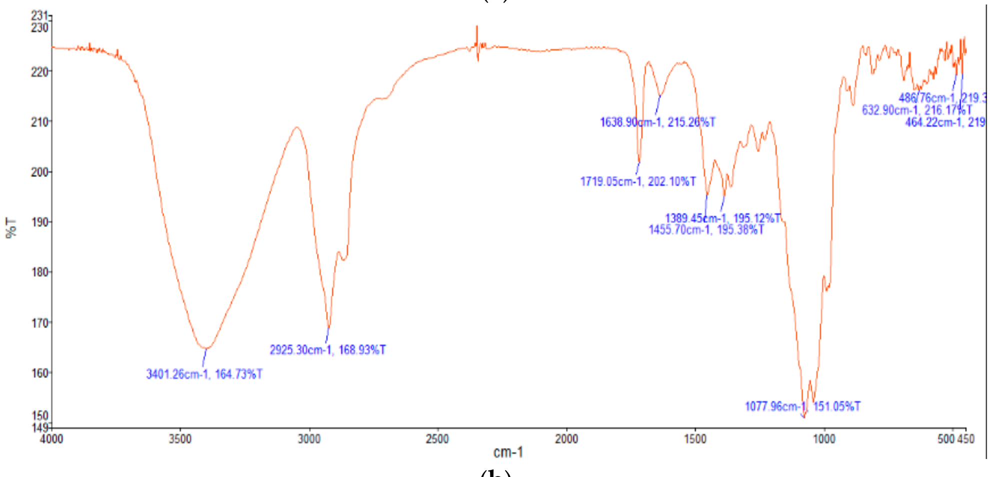

(**b**)

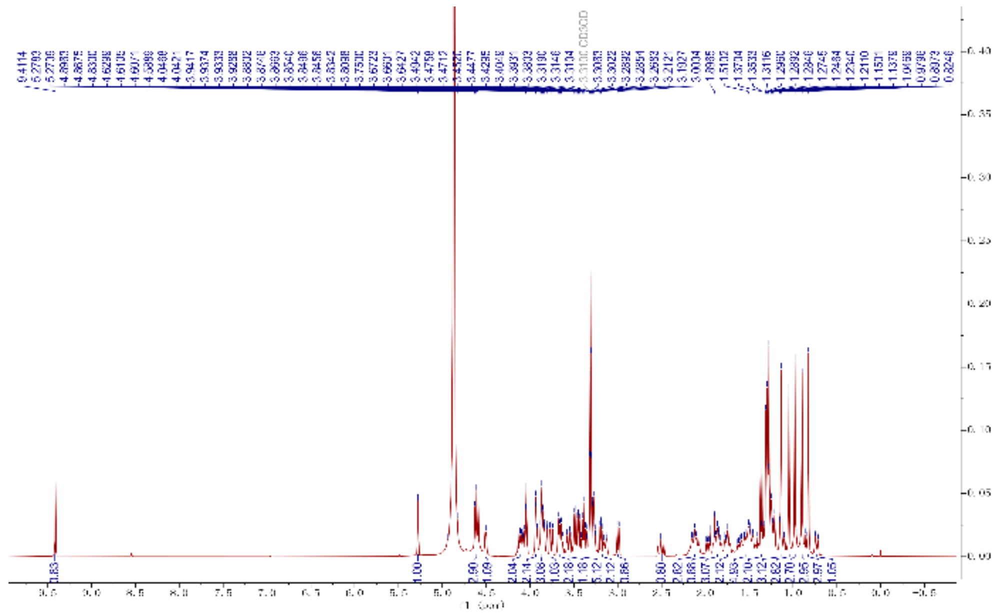

(**c**)

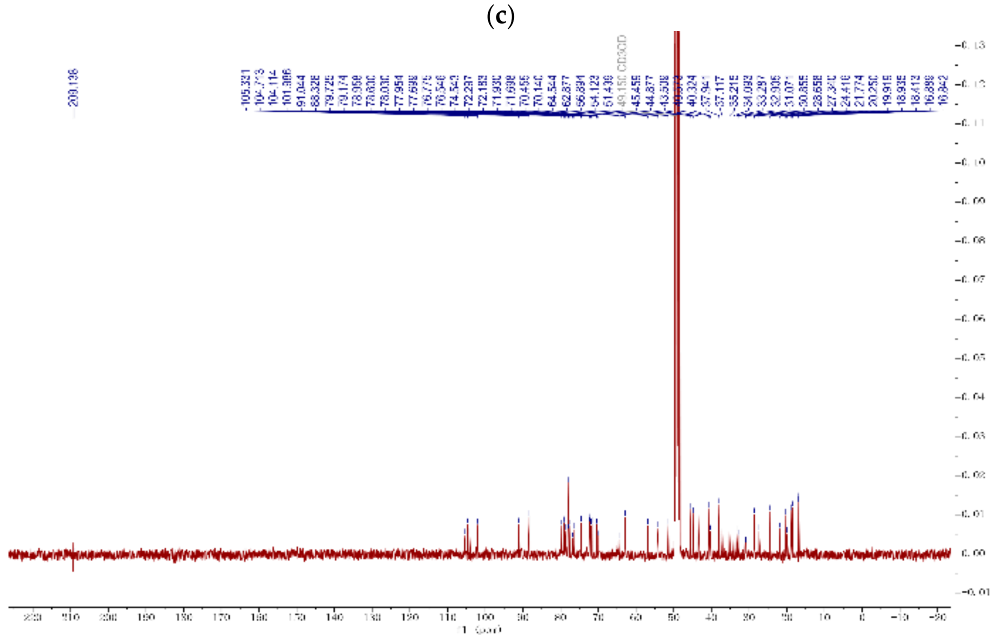

(**d**)

Figure s2. Characterization information of ardisiacrispin B compounds obtained by isolation: (**a**) chemical structure, (**b**) infrared spectrum, (**c**) ^{1}H NMR, (**d**) ^{13}C NMR.

Table S1 Ardisiacrispin B targets related to inflammation

Target name									
ABCB1	ABCB11	ABCG2	ADORA1	ADORA2A	ADORA3	AGTR1	AGTR2	BACE1	CASP1
CASP3	CASP9	CCKBR	CNR1	CNR2	CTSD	CYP2D6	DRD2	FDFT1	FGF1
FGF2	GLRA1	GRB2	HDAC1	HDAC6	HLA-A	HPSE	HSD11B2	HSP90AA1	HTR1B
IKBKB	IL2	ITGA2B	ITGA4	ITGAV	ITGB1	ITGB3	ITGB6	LCK	LGALS3
LGALS4	LGALS8	LIPC	LIPG	LTB4R	MMP1	MMP2	MMP3	MMP7	NCSTN
OPRD1	OPRK1	OPRM1	P2RY12	PIM1	PPIA	PSEN1	PSENEN	PTAFR	PTPN11
PTPN2	PTPN22	PTPRC	RORC	STAT3	TACR1	TBXA2R	TOP1	TRPV1	TRPV4
VDR	VEGFA	XIAP							

Table S2 Metabolites targets related to inflammation

Target name								
ABCB1	ABL1	ADORA1	ADORA2A	ALK	ALOX5	ANPEP	APP	AR
ATP12A	AURKA	BACE1	BCL2L1	BRAF	BRD4	C5AR1	CA2	CCR1
CDK2	CDK5	CDK9	CHEK2	CNR1	CTRC	CTSD	CTSS	CXCR3
CYP19A1	CYP24A1	CYP2C9	CYP2D6	DNMT1	DPP4	DRD2	EGFR	ENPP2
EPHX2	ESR2	F10	F2	F2R	FAAH	FABP4	FASN	FDFT1
FFAR1	FGF1	FGF2	FLT1	FLT4	GBA	GLB1	GLRA1	GSK3A
GSK3B	HMGCR	HPSE	HRH1	HSD11B1	HSD11B2	HSD3B1	HSP90AA1	HSP90AB1
HSP90B1	HTR1B	ICAM1	IGF1R	IL6ST	INSR	ITGAL	ITGB2	ITK
JAK1	JAK2	JAK3	KCNK3	KDR	LDLR	LGALS3	LGALS4	LGALS8
LIMK1	LRRK2	LTB4R	MAPK1	MAPK10	MAPK14	MAPK8	MAPK9	MAPKAPK2
MC4R	MDM2	MET	MMP1	MMP2	MMP3	MMP7	MTOR	NCSTN
NLRP3	NOS2	NR1H2	NR1H3	NR1H4	NR1I2	NR1I3	NR3C1	NR3C2
OPRD1	OPRK1	OPRM1	P2RX3	PARP1	PDE3B	PDGFRB	PGR	PIK3CA
PIK3CB	PIK3CD	PIK3CG	PLA2G4A	PLA2G7	PNP	POLA1	PPARA	PPARD
PPARG	PPP1CA	PRKCA	PRKCD	PRKCE	PRKCG	PRKCQ	PSEN1	PSENEN
PTAFR	PTGES	PTGS1	PTGS2	PTPN1	PTPN2	REN	RHOA	RORC
S1PR1	S1PR3	SCN9A	SERPINA6	SHBG	SLC5A2	SMO	SRD5A1	SRD5A2
STAT3	TACR1	TACR2	TBXA2R	TEK	TRPV1	TRPV4	TSPO	TYK2
VDR	VEGFA							
